# Prognosticating Outcome in Pancreatic Head Cancer With the use of a Machine Learning Algorithm

**DOI:** 10.1177/15330338211050767

**Published:** 2021-11-05

**Authors:** Zarrukh Baig, Nawaf Abu-Omar, Rayyan Khan, Carlos Verdiales, Ryan Frehlick, John Shaw, Fang-Xiang Wu, Yigang Luo

**Affiliations:** 1 7235 University of Saskatchewan, Saskatoon, Canada; 2 12371 College of Medicine, 7235 University of Saskatchewan, Saskatoon, Canada

**Keywords:** pancreatic cancer, machine learning, supervise learning model, prognosis, whipple procedure, pancreaticoduodenectomy

## Abstract

**Background:** The purpose of this project is to identify prognostic features in resectable pancreatic head adenocarcinoma and use these features to develop a machine learning algorithm that prognosticates survival for patients pursuing pancreaticoduodenectomy. **Methods:** A retrospective cohort study of 93 patients who underwent a pancreaticoduodenectomy was performed. The patients were analyzed in 2 groups: Group 1 (n = 38) comprised of patients who survived < 2 years, and Group 2 (n = 55) comprised of patients who survived > 2 years. After comparing the two groups, 9 categorical features and 2 continuous features (11 total) were selected to be statistically significant (p < .05) in predicting outcome after surgery. These 11 features were used to train a machine learning algorithm that prognosticates survival. **Results:** The algorithm obtained 75% accuracy, 41.9% sensitivity, and 97.5% specificity in predicting whether survival is less than 2 years after surgery. **Conclusion:** A supervised machine learning algorithm that prognosticates survival can be a useful tool to personalize treatment plans for patients with pancreatic cancer.

## Introduction

Pancreatic ductal adenocarcinoma (PDAC) has a poor prognosis with an overall five-year survival of only 8%.^1^ It is the fourth leading cause of cancer related mortality in North America.^2^ Symptoms are vague and non-specific until advanced stages of the disease.^3^ Surgical resection is the only potential curative treatment for PDAC but given the frequently delayed presentation, only 15 to 20% of patients are considered surgical candidates at the time of diagnosis.^2,4^

Pancreaticoduodenectomy, more commonly referred to as the Whipple procedure, is the surgical treatment of choice in patients who have a resectable tumour in the pancreatic head and are surgically fit.^5^ Approximately 60 to 70% of pancreatic adenocarcinomas are located in the head of pancreas.^6^ Tumours are determined to be resectable based on the involvement of nearby structures (superior mesenteric artery and vein, portal vein, hepatic artery, and celiac artery); in addition to the absence of distance metastasis and overall surgical fitness of patients.^7^ Despite the significant improvements in peri-operative mortality that are down to 2 to 3%, this remains a challenging surgical procedure with a high morbidity of up to 40 to 50%.^7^ Complications following pancreaticoduodenectomy can drastically affect a person's quality of life. These include delayed gastric emptying, new-onset diabetes, intra-abdominal infections, pancreatic fistulas, pancreatic insufficiency, hemorrhage, and death.^8^ Therefore, it is imperative to identify prognostic factors that aid in the selection of good surgical candidates and limit patients with an expected poor outcome from undergoing a major surgery.

Traditionally, TNM (Tumor, lymph Node, Metastasis) staging is used to predict patient survival in majority of cancers. However, not all information for TNM staging can be accurately available before the final pathology, often from a surgery. Like most centers, we often use tumour staging (resectable, borderline resectable and unresectable) to determine if patients should undergo surgery. However, an assessment of resectability is not an accurate prognosticator for survival after surgery. It is simply a means to determine if surgery is feasible. In fact, patients with borderline resectable and resectable cancer can have similar prognosis, and within resectable cancers, patients are heterogeneous, carrying different prognosis.^9,10^ This prognostic strategy lacks patient specific characteristics and offers limited benefit to patients who are deciding whether to pursue surgery. Several studies have analyzed characteristics that prognosticate survival in pancreatic cancer with variable findings.^11,12^ Despite the evidence to support these prognostic factors, their use in clinical practice is still limited as there is no statistical tool that incorporates all discriminate factors and estimates survival. Therefore, it would be of immense value to have an algorithm that analyzes all the prognostic factors to estimate an overall survival. This would be helpful in guiding individualized patient management plans with a focus on overall survival and quality of life.

There has been a growing trend that favours the utilization of computational algorithms to assist in medical decision making. To individualize treatment plans, several online prognostic calculators have been developed for various malignancies.^13,14^ These calculators analyze patient specific prognostic factors to estimate overall survival and recommend the best management strategy. Previously, prognostic algorithms used mathematical models that relied on multivariate regression and Bayesian models.^13,14^ The University of Texas has created an online calculator that estimates conditional survival for patients with pancreatic cancer using a regression model.^15^ This allows for determining prognosis at unique time points in a patient's lifetime. The University of Iowa has similarly created an online calculator that predicts outcome after pancreaticoduodenectomy based on the lymph node status using a Bayesian model.^16^ Additionally, Bradley et al (2019) published an algorithm that prognosticates survival based on pre-operative findings using a Bayesian model.^17^ Recent literature has demonstrated the value of using Machine Learning (ML) algorithms for prognosticating the progression of various diseases as well as the treatment efficacy.^18^ These studies have shown that machine learning can offer more accurate results for predicting outcome compared to traditional Bayesian and Regression models.^17,18^

To the best of our knowledge, this is the first study to develop a supervised machine learning algorithm for prognosticating survival in pancreatic cancer patients undergoing pancreaticoduodenectomy. It is also the first study that utilizes multiple feature selection techniques such as statistical significance, recursive feature elimination, and stable selection methods to optimize prognostic factors, and use the selected features to train a machine learning algorithm.

## Materials and Methods

This was a retrospective cohort study of 113 patients with pancreatic head adenocarcinoma that underwent a pancreaticoduodenectomy in Saskatoon, Canada from 2009 to 2019 was performed. In total, there was a larger volume of whipples performed in Saskatoon during that time, but our study was limited to pancreatic head ductal adenocarcinoma, which restricted our sample size to 113 patients. In our center, patients are selected for surgery if they fit into the resectable or borderline resectable criteria, in conjunction with multidisciplinary team discussions. This frequently corelates with TNM stages I, II, and III, however, the final pathological stage cannot be confirmed prior to surgery. This study was approved by the University of Saskatchewan Research Ethics Board, Saskatoon, Canada (Bio 807 -June second 2019). In keeping with the Enhancing the Quality and Transparency Of health Research (EQUATOR) guidelines, the reporting of this study conforms to the STROBE checklist for retrospective cohort studies.^19^

Characteristics such as tumour pathology, patient comorbidities, clinical presentation, laboratory and molecular factors, and adjuvant treatment were assessed. To include all potential prognostic factors, we selected 50 variables that could theoretically affect survival in pancreatic cancer (Table 1). The selection of factors was based on the data available in our electronic medical records (EMR). Some of the newly recognized prognostic factors such as p53 or BRCA could not be included due to their lack of availability. However, several factors were included that are not established in the literature with the presumption that machine learning algorithms could identify novel prognostic factors.

**Table 1.** List of 50 potential factors that could affect survival after pancreaticoduodenectomy selected by a team of Hepatopancreaticobiliary Surgeons in Saskatoon. These factors were then subsequently analyzed to determine 11 specific features that can predict survival < 2 years. List of abbreviations: ASA (American Society of Anesthesiologists classification), T2DM (Type 2 Diabetes Mellitus), HTN (Hypertension), CAD (Coronary Artery Disease), AFP (alpha fetoprotein), CA19 to 9 (Carbohydrate Antigen 19-9 tumour marker), ERCP (Endoscopic Retrograde Cholangiopancreatography Procedure), CT (Computerized Tomography), MRI (Magnetic Resonance Imaging), CBD (Common Bile Duct), SMA (Superior Mesenteric Artery), and SMV (Superior Mesenteric Vein).

Demographics:	Gender	Age	Type pf Cancer	Family History of Caner	Type of Familial Cancer		
Comorbidities:	BMI	ASA	T2DM	HTN	CAD	Dyslipidemia	MEN 1 mutation
	Smoking	Alcohol					
Presenting Symptoms:	New Diagnosis of DM	Abdominal/Back Pain	Jaundice	Weight loss (> 15 lb)			
Laboratory Investigations:	Total Bilirubin	Lipase	Albumin	Pre-albumin	AFP	CA19 - 9	
Histopathology:	T-Stage	N-stage	M-Stage	Pathological Differentiation	Additional pathology	Tumor Site and Invasion	Tumor size on pathology
	Dysplasia of Portal Tissue						
Imaging:	ERCP	Tumor size -CT	Tumor Size- MRI	CBD Dilation	CBD Stricutre	Pancreatic Duct Dilation	SMA Involvement
	SMV Involvement	Portal Vein Involvement	Perineural Involvement	Other Involvement	SMV Involvement		
Surgical Resection:	Margin (P or N)	Portal Vein Resection					
Other Treatments & Outcome:	Neoadjuvant Therapy	Adjuvant Therapy	Hospital Stay (d)	Post-op Complications	Recurrence	Site of Recurrence or Metastasis	

**Figure 1.**
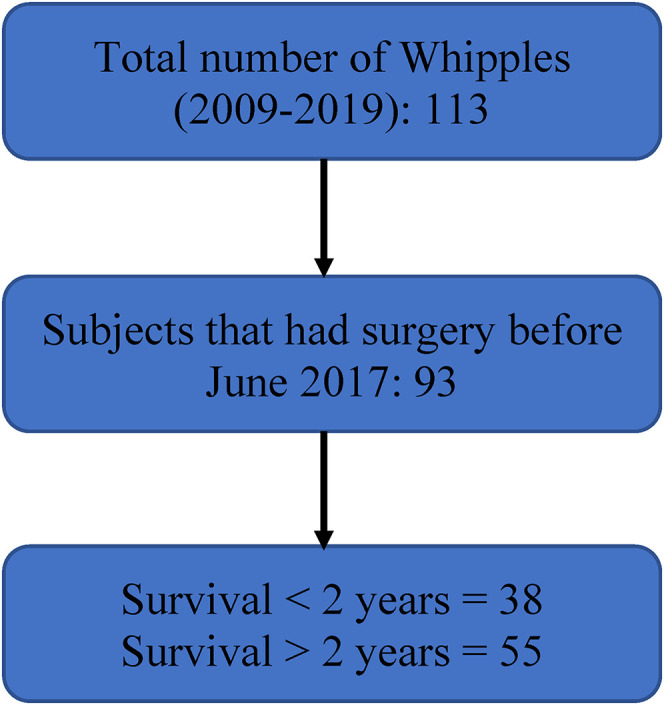
Selection of participants after pancreaticoduodenectomy for PDAC.

Twenty patients who had surgery after June 2017 were excluded from analysis to allow at least 2 years of follow-up data post-surgery. This also allowed us to classify all patients under the seventh edition of the TNM staging for pancreatic cancer. The remaining 93 patients (Male = 52, Female = 41) were analyzed in 2 groups: Group 1 (n = 38) comprised of patients who had survival < 2 years, and Group 2 (n = 55) comprised of patients who had survival > 2 years (Figure 1). Due to the limited number of patients in the study, we divided our patients into two relatively large groups, patients surviving less than 2 years (Group 1) and surviving more than 2 years (Group 2). Ideally, we would have grouped patients in shorter intervals. For instance, patients with survival <6 months may not pursue surgery due to the minimal improvement in their survival with surgery and thereby benefit the most from the algorithm. However, this was not feasible due to the number of patients required in each category to train a valid model.

Using three different selection techniques 11 out of 50 unique prognostic factors (9 categorical and 2 continuous) were selected to be optimal in predicting survival < 2 years and > 2 years after surgery. Table 2 provides an overview of the selected factors used to prognosticate pancreatic adenocarcinoma after surgery. These 11 selected factors were then used to train a nonlinear Support Vector Machine (SVM) algorithm that could prognosticate overall survival.

**Table 2.** 11 prognostic factors that predict survival in pancreatic ductal adenocarcinoma. These were selected using 3 separate statistical tests that included statistical significance testing, recursive feature elimination, and stable selection techniques.

Prognostic Factors (Mean ± SD)		
Variables	Survival < 2 Years	Survival > 2 Years
**Categorical Factors**		
T2DM	0.487 ±0.506	0.272 ± 0.449
FHx of Cancer	0.315 ± 0.471	0.527 ± 0.503
Number of Family Members with Cancer	0.324 ± 0.474	0.545 ± 0.502
Bile duct Stricture	0.315 ± 0.471	0.145 ± 0.355
Perineural involvement	0.736 ± 0.446	0.309 ± 0.466
Margins (positive)	0.605 ± 0.495	0.218 ± 0.416
Portal vein tissue resection	0.447 ± 0.503	0.072 ± 0.262
Neoadjuvant therapy	0.081 ± 0.276	0.076 ± 0.269
Adjuvant therapy	0.815 ± 0.392	0.962 ± 0.192
**Continuous Factors**		
Size of tumor based on MRI	1.797 ± 1.327	2.725 ± 2.125
Size of tumor based on pathology	1.793 ± 1.054	1.574 ± 1.319

To initially select prognostic factors from categorical features, we used a binomial-test for two proportions to identify prognostic factors that were significantly different between group 1 (survival < 2 years) and group 2 (survival > 2 years) (see Appendix; Equation 1). This resulted in 8 categorical features that were significantly different between the two groups at a significance level of p <.05.^20,21^ To select prognostic factors from continuous features, a two-sided student's t-test was utilized. This resulted in 1 prognostic factor, tumour size on MRI, that was statistically significant between group 1 and 2 at p <.05.(see Appendix; Equation 2)^22,23^

In human physiology, certain prognostic factors have a degree of interdependence such as the presence of obesity, diabetes, and back pain. Since the purpose of employing a ML algorithm was to incorporate all prognostic factors that estimated overall survival, it was imperative to identify any ‘interdependent prognostic factors' even if they are not statistically significant independent prognosticators of survival. Thus, we applied a second feature selection technique, a ‘recursive feature elimination’ with greedy optimization to exclude the weakest prognostic factors based on model cross-validation scores. The weakest features were rejected by fitting a nonlinear SVM model multiple times and at each step removing the weakest feature. This process was repeated until the model cross-validation score was increased to the highest possible score. The recursive feature elimination resulted in finding three additional prognostic factors (2 categorical and 1 continuous): use of neoadjuvant therapy, adjuvant therapy, and the size of the tumour based on pathology.

Finally, the new subset of features from the recursive feature selection were then combined with the previously obtained features from the initial statistical tests to perform ‘stable selection’ based on subsampling of features in combination with the selection algorithm. To conclude with the best subsample of prognostic factors, we evaluated the proposed model with all possible combinations and then calculated cross-validation scores with the average of multiple runs, and selected the factors with the highest cross-validation scores for the algorithm. In summary, we applied three separate methods to identify 11 optimal prognostic factors out of 50. Nine out of these 11 features were common between statistical selection and recursive feature elimination methods.

In this study, a supervised learning algorithm was trained given the low volume of available data. Within supervised learning algorithms, the nonlinear SVM model has been shown to have the best prediction capabilities among all the supervised machine learning algorithms.^22^ Therefore, it is best suited for the purposes of determining prognosis in pancreatic cancer, with a future role of developing a prognostic calculator for selecting surgical candidates. We also performed a comparative analysis of 3 commonly used algorithms. In Table 3 the Naïve Bayes, Random Forest, and SVM models are compared based on their Leave One Out Cross-Validation (LOOCV) scores and testing accuracies. The random forest regression model is applied using entropy criterion with ten estimators. Please refer to the Appendix for detailed information on the Naïve Bayes and Random Forest analysis. Based on these scores, the SVM model was used given its high testing accuracies and specificity.

**Table 3.** Comparison of various machine learning methods and their respective performances. In this study, an SVM algorithm was utilized given it provided the highest testing accuracy.

Classifier	Training	Testing
SVM	0.7527	0.7526
Random Forest	0.6844	0.6733
Naïve Bayes	0.7526	0.7469

Once the 11 features were identified, the nonlinear SVM algorithm was trained to predict outcome between the two groups (survival < 2 years and survival > 2 years). The nonlinear SVM was utilized to classify between two class data with a sigmoid function and regularization parameter of C=0.1 (see Appendix; Equation 3).^24,25^ Owing to the limitation of our data, the Leave One Out Cross-Validation (LOOCV) was preferred over k-fold cross-validation to illustrate the performance of our model. The model was first evaluated based on the LOOCV and testing accuracies with an average of multiple runs (see Appendix; training and validating the SVM model). Finally, test specificity and sensitivity were obtained by dividing the data set into 90% for training and 10% for testing the data (Table 4).

**Table 4.** Testing data confusion matrix. Based on the confusion matrix, this algorithm has a sensitivity of 41.9% and specificity of 97.5% in predicting survival < 2. The algorithm was designed using 90% of the data. 10% of the data was used for testing the sensitivity and specificity of the algorithm. The model was also cross-validated using the entire subset of 93 subjects by applying 300 runs of the leave-one-out cross validation method. This achieved a mean training accuracy of 75.26%.

		**Predicted groups based on algorithm**		
		Survival < 2 years	Survival > 2 years	
**Actual Values (10% testing data)**	Survival < 2 years (n = 38) 4 (10% of 38)	TP = 1.6766	FN = 2.3233	Sensitivity = 41.9%
	Survival > 2 years (n = 55) 6 (10% of 55)	FP = 0.150	TN = 5.850	Specificity = 97.5%
		2.756	7.242	ACC = .7526

## Results

Using categorical data, we found patients in group 1 who had survival < 2 years following surgery had significantly increased (p < .05): type 2 diabetes, familial history of cancer, increased number of family members with cancer, bile duct stricturing, perineural involvement of tumour, positive resection margin, resection of portal vein in surgery, and recurrence of cancer. For continuous data, we found the tumor size on MRI to be significantly increased in group 2 (survival > 2 years). In addition, after applying a recursive elimination method and stable selection model to obtain interdependent characteristics, we also found that adjuvant chemotherapy, neoadjuvant chemotherapy, and tumour size on pathology were predictors of improved outcome in group 2 (survival > 2 years). This resulted in 12 prognostic factors in total, however recurrence was removed from the SVM model given that it was a post-operative prognostic factor, and unlikely to be available for all patients.

Once the 11 factors were selected, a SVM algorithm was trained to create a machine learning model. The model was validated in two sequential analyses. First, the model was cross-validated using the entire subset of 93 subjects by applying 300 runs of the leave-one-out cross validation method. This achieved a mean training accuracy of 75.27%. Next, the algorithm was assessed by dividing the subjects into 90% for training the algorithm and 10% for testing. This model achieved a sensitivity of 41.9%, and specificity of 97.5% in predicting survival. Out of 10 random test patients the trained model, on average, predicted 7.526 patients (75%) correctly (Table 4). Since the testing accuracy [75.26%] is close to the leave-one-out mean accuracy [75.27%], we can confirm an optimal performance by this algorithm. The algorithm enables clinicians to input the 11 prognostic factors and predict overall patient survival with an accuracy of 75% and specificity of 98%.

## Discussion

Pancreatic ductal adenocarcinoma (PDAC) has a poor survival even with surgery for curative intent. Pancreaticoduodenectomy is the only potential curative treatment for patients. This surgery carries significant morbidity. Therefore, it is important to be selective of patients considered for surgery. Many prognostic factors have been identified over the years, and collectively, these prognostic factors can aid patients and clinicians in choosing treatment options that focus on patients having the best quality of life.^26,27^

Using three separate selection techniques, we have identified 9 categorical factors and 2 continuous factors that were statistically significant (P < .05) in prognosticating survival < 2 years following surgery (Table 2.). These included patient background health factors, tumor biology factors and treatment factors. These 11 features were used to train a Support Vector Model (SVM) algorithm that could prognosticate survival after pancreaticoduodenectomy. The algorithm obtained 75% testing accuracy in predicting survival < 2 years or > 2 years.

Overall, our identified factors concur with the previously published data for prognosticating pancreatic cancer with the additional findings of familial history of any cancer, number of family members with any cancer, and findings of bile duct stricturing.^28-31^ We believe that familial history of cancer and number of family members diagnosed with cancer indicate that such patients have a genetic predisposition to malignancy and are perhaps more likely to succumb to pancreatic cancer. Bile duct stricturing associated with jaundice matches the literature findings of preoperative jaundice being a poor prognostic factor in pancreatic cancer.^28^ An additional factor that we discovered correlating with survival < 2 years was the recurrence of cancer itself, which was subsequently removed as it was not available for all patients. Recurrence is obviously a major cause of death in pancreatic cancer and is well established in the literature, but it offers little value for patients deciding whether to pursue pancreaticuoduodenectomy.^32^

Using the 11 identified factors, we developed a SVM algorithm to prognosticate outcome. To the best of our knowledge, there are currently no prognostic algorithms that utilize supervised machine learning models in predicting outcome in pancreatic cancer after a pancreaticoduodenectomy. To cross-validate the results of our study, we also performed a classical multivariate regression analysis. Prognostic factors were first identified using a univariate analysis, and variables with p-values <.05 were used in the multivariate regression analysis. Three variables were shown to have an odds ratio greater than 1 for survival of < 2 years. These were bile duct stricturing, positive surgical margins on pathology and portal vein resection in surgery. These variables correlated with the previous literature and our algorithm findings. However, our SVM model identified 11 factors compared to these 3 identified using multivariate regression. Our study therefore supports the notion that ML algorithms outperform traditional prognostic models developed using regression.

In the past, several studies have tried to estimate accurate prognostic factors using various mathematical schemes mainly based on multivariate regression and Bayesian models.^11,13,14,18,33^ Regression algorithms are predictive modelling techniques that define the relationship between dependent and independent variables. Bayesian techniques rely on a probabilistic model based on the statistical distribution of the data. These statistical-based techniques are often used for prognostication even though these models require assumptions of independent observations, linearity of data, and normal distribution. Machine Learning models do not require these assumptions. Machine Learning is a growing area in medicine that uses distinct dependent attributes to predict outcomes. Recent literature has demonstrated the value of using Machine Learning (ML) algorithms for prognosticating disease progression and treatment efficacy.^18^ In patients with pancreatic cancer, the clinical data is usually nonlinear with distinct features, and with interdependence of lab findings and other prognostic factors. As such, we believe that machine learning algorithms can make more accurate predictions compared to traditional prognostic models.^22,24,25^ Thus, in this study, we applied a nonlinear Support Vector Machine (SVM) model to develop a prognostic algorithm for resectable pancreatic cancer. Based on a comparative analysis of different models (Table 3), the SVM model was selected because of its high testing accuracies and specificity. For factor selection, various techniques have been adopted in previous studies.^11,21,34^ We applied a combination of three separate techniques to obtain the most optimal factors that would subsequently be used to train the SVM model. To the best of our knowledge, a supervised ML algorithm with optimal feature selection has not been previously used to prognosticate survival in pancreatic cancer. Hence, the purpose of this project was to 1) identify prognostic features in pancreatic cancer using 3 different factor selection techniques and 2) use the identified factors to develop a SVM algorithm that prognosticates survival <2 years versus > 2 years. The trained algorithm from this study can enable clinicians to input the 11 identified prognostic factors and predict overall patient survival with an accuracy of 75% and specificity of 98%. This can make a profound impact for patients and clinicians determining whether to pursue a pancreaticoduodenectomy.

There are some limitations to this study. Most of these are attributed to small number of patients in the study, and that is a direct reflection of the overall population in Saskatchewan. Furthermore, our Research Ethics Board (REB) guidelines restrict data collection to a maximum of 10 years. One of the major restrictions of the study is that it predicts survival less than 2 years and more than 2 years. These groups have a range of 0 to 24 months and more than 24 months. Preferably, we would include shorter intervals such as survival <6 months as that would provide us with more specific patient cohorts. However, this was not feasible due to the limited study population as the algorithm requires a similar number of patients in each category.

Additionally, 5 prognostic factors were postoperative, which was counterintuitive to our initial goal of determining prognosis using pre-operative prognostic algorithm. The expectation of the study originally was to determine pre-operative prognostic factors that could then predict patient outcome prior to surgery. Unfortunately, the ML algorithm yielded a significant number of post-operative factors to meet optimal capabilities, due to the limited number of patients in the study. However, using both pre-operative and post-operative prognostic factors still allows us to determine patient's overall survival, albeit after surgical intervention. In the future, we would like to develop a prognostic algorithm using only pre-operative prognostic factors through a multi-institution collaboration.

There are potential biases in the algorithm design. The same cohort that was used to identify prognostic factors was used to train the ML algorithm potentially amplifying any biases. This also applies to leave-one-out cross validation. However, we tried to circumvent this bias by utilizing 90% of the cohort for training the model, and 10% of the cohort to validate it (Table 4). We understand that this current model is not clinically applicable at present, but it demonstrates the feasibility of developing a robust algorithm that would be generalizable to an independent data set. This trained machine learning model may have great potential when large data sets become available.

## Conclusion

In this study we utilized three distinct tests to select 11 prognostic features that were used to optimize the two-class nonlinear SVM model. The trained algorithm was able to achieve 75% mean accuracy for test data that is close to the validation mean accuracy (achieved through LOOCV). It can therefore be concluded that the trained algorithm can prognosticate survival in patients with pancreatic adenocarcinoma. Further data is required to optimize the algorithm and predict survival based on separate pre-operative and post-operative factors.

The utilization of machine learning algorithms is a growing area in the field of medicine. Using a machine learning algorithm that can accurately prognosticate outcome is a useful tool to individualize treatment plans for patients with pancreatic cancer. It will help prevent futile surgery and focus on treatment that achieves the best outcome.

## Supplemental Material

sj-docx-1-tct-10.1177_15330338211050767 - Supplemental material for Prognosticating Outcome in Pancreatic Head Cancer With the use of a Machine Learning Algorithm Click here for additional data file. Supplemental material, sj-docx-1-tct-10.1177_15330338211050767 for Prognosticating Outcome in Pancreatic Head Cancer With the use of a Machine Learning Algorithm by Zarrukh Baig, Nawaf Abu-Omar, Rayyan Khan, Carlos Verdiales, Ryan Frehlick, John Shaw, Fang-Xiang Wu and Yigang Luo in Technology in Cancer Research & Treatment

sj-docx-2-tct-10.1177_15330338211050767 - Supplemental material for Prognosticating Outcome in Pancreatic Head Cancer With the use of a Machine Learning Algorithm Click here for additional data file. Supplemental material, sj-docx-2-tct-10.1177_15330338211050767 for Prognosticating Outcome in Pancreatic Head Cancer With the use of a Machine Learning Algorithm by Zarrukh Baig, Nawaf Abu-Omar, Rayyan Khan, Carlos Verdiales, Ryan Frehlick, John Shaw, Fang-Xiang Wu and Yigang Luo in Technology in Cancer Research & Treatment

sj-pdf-3-tct-10.1177_15330338211050767 - Supplemental material for Prognosticating Outcome in Pancreatic Head Cancer With the use of a Machine Learning Algorithm Click here for additional data file. Supplemental material, sj-pdf-3-tct-10.1177_15330338211050767 for Prognosticating Outcome in Pancreatic Head Cancer With the use of a Machine Learning Algorithm by Zarrukh Baig, Nawaf Abu-Omar, Rayyan Khan, Carlos Verdiales, Ryan Frehlick, John Shaw, Fang-Xiang Wu and Yigang Luo in Technology in Cancer Research & Treatment

sj-docx-5-tct-10.1177_15330338211050767 - Supplemental material for Prognosticating Outcome in Pancreatic Head Cancer With the use of a Machine Learning Algorithm Click here for additional data file. Supplemental material, sj-docx-5-tct-10.1177_15330338211050767 for Prognosticating Outcome in Pancreatic Head Cancer With the use of a Machine Learning Algorithm by Zarrukh Baig, Nawaf Abu-Omar, Rayyan Khan, Carlos Verdiales, Ryan Frehlick, John Shaw, Fang-Xiang Wu and Yigang Luo in Technology in Cancer Research & Treatment

## Abbreviations

BUN: Blood Urea Nitrogen
BMI: Body Mass Index
BRCA: Breast Cancer Gene
CA 19 to 9: Carbohydrate Antigen 19 to 9
COPD: Chronic Obstructive Pulmonary Disease
EMR: Electronic Medical Record
LDH: Lactate Dehydrogenase
LVOOCV: Leave One Out Cross-Validation
ML: Machine Learning
MRI: Magnetic Resonance Imaging
PDAC: Pancreatic Ductal Adenocarcinoma
SVM: Support Vector Machine
TNM: Tumor, Node, Metastasis.
