# Supplementary material for: Prognosticating Outcome in Pancreatic Head Cancer With the use of a Machine Learning Algorithm
Source: Technol Cancer Res Treat. 2021 Nov 5;20:15330338211050767. doi: 10.1177/15330338211050767 (PMC8573477; doi:10.1177/15330338211050767)
Supplement: sj-docx-1-tct-10.1177_15330338211050767 - Supplemental material for Prognosticating Outcome in Pancreatic Head Cancer With the use of a Machine Learning Algorithm [file sj-docx-1-tct-10.1177_15330338211050767.docx]

**Pancreatic Cancer SVM Model-Statistics Explained**

Total Subjects: 113

Deceased Subjects: 55

Alive Subjects: 58

Subjects that had surgery before June 2017 = 38

Subjects that had surgery after June 2017 = 20

Hence 93 subject's data were used for analysis.

1. **Selecting Significant Features for the SVM Model**

**Feature Selection with Statistical Tests for Parametric Data**

Data consist of binary and continuous data therefore, we applied two different Statistical tests to get significant features

**Equation 1. Signiﬁcance Test for Binomial data with two Proportions**

We performed null hypothesis test based on two independent populations (Pagano and Gauvreau, 2018). Such that,

$$H_{o}:p_{1}=p_{2}; H_{A}:p_{1}\neq p_{2}$$

To conduct test, we draw sample of size $n_{1}$ from the population with mean $p_{1}$ and sample of size $n_{2}$ from the population with mean $p_{2}$. Let the number of successes in both samples be $x_{1}$ and $x_{2}$, then

$$\hat{p_{1}}=\frac{x_{1}}{n_{1}}, \hat{p_{2}}=\frac{x_{2}}{n_{2}}$$

To determine whether the difference in sample proportions $(\hat{p_{1}}-\hat{p_{2}})$ is too large, we compute the probability of obtaining a pair of proportions different, given null hypothesis is true. If this probability is sufficiently small, then we reject the null hypothesis.

Thus, considering null hypothesis is true, then the population proportion are equal given as,

$$\hat{p}=\frac{x_{1}+x_{2}}{n_{1}+n_{2}}$$

Under the null hypothesis, the standard error of the difference $(p_{1}-p_{2})$ takes the form, $\sqrt{\hat{p}\left( 1-\hat{p} \right)[(\frac{1}{n_{1}})+(\frac{1}{n_{2}})]}$

The test statistics become,

$$z=\frac{(\hat{p_{1}}-\hat{p_{2}}){-(p}_{1}-p_{2})}{\sqrt{\hat{p}\left( 1-\hat{p} \right)[(\frac{1}{n_{1}})+(\frac{1}{n_{2}})]}}$$

Provided test statistics follow normal distribution, we can compute p-values for two-sided test to select features at significance level of $\alpha=0.05$.

**Equation 2. Signiﬁcance Test for Continuous Data - Student's t-test**

Since, we have two independent small samples data, we conducted null hypothesis test based on student's t distribution at $\alpha=0.05$ level of significance. Such that (Navidi, 2008),

$$H_{o}:\mu_{X}=\mu_{Y}; H_{A}:\mu_{X}\neq\mu_{Y}$$

The test statistic is given by,

$$t=\frac{\left( \bar{X}-\bar{Y} \right)-(\mu_{X}-\mu_{Y})}{\sqrt{{s_{X}^{2}}/{n_{X}+{s_{Y}^{2}}/{n_{Y}}}}}$$

where, $(\bar{X}\&\bar{Y}$) are samples mean in each category, $(s_{X}\&s_{Y})$ are standard deviation, $(\mu_{X}\&\mu_{Y})$ are true difference population means for the null hypothesis and $(n_{X}\&n_{Y})$ are two sample sizes.

The degree of freedom $(v)$ of this student's t distribution is given by,

$$v=\frac{\left[ {s_{X}^{2}}/{n_{X}+{s_{Y}^{2}}/{n_{Y}}} \right]^{2}}{\frac{\left[ {s_{X}^{2}}/{n_{X}} \right]^{2}}{n_{X}-1}+\frac{\left[ {s_{Y}^{2}}/{n_{Y}} \right]^{2}}{n_{Y}-1}}$$

It is rounded down to nearest integer.

**Python implementation**

**scipy.stats.ttest_ind(***a***,***b***,***axis=0***,***equal_var=False***,***nan_policy='propagate'***)**[**[source]**](https://github.com/scipy/scipy/blob/v1.3.1/scipy/stats/stats.py#L4450-L4572)

Calculate the T-test for the means of *two independent* samples of scores.

This is a two-sided test for the null hypothesis that 2 independent samples have identical average (expected) values. This test assumes that the populations have identical variances by default but can be changed to unequal variances.

**Parameters**

**a, b *array_like***

The arrays must have the same shape, except in the dimension corresponding to *axis* (the first, by default).

**Axis *int or None, optional***

Axis along which to compute test. If None, compute over the whole arrays, *a*, and *b*.

**equal_var *bool, optional***

If True (default), perform a standard independent 2 sample test that assumes equal population variances. If False, perform Welch's t-test, which does not assume equal population variance.

**nan_policy *{‘propagate’, ‘raise’, ‘omit’}, optional***

Defines how to handle when input contains nan. 'propagate' returns nan, 'raise' throws an error, 'omit' performs the calculations ignoring nan values. Default is 'propagate'.

**Returns**

**statistic*float or array***

The calculated t-statistic.

**pvalue*float or array***

The two-tailed p-value.

**Feature Selection with recursive feature elimination and stable selection**

We also applied greedy optimization technique to exclude weakest features first based on model cross-validation score. The weakest out of $n$ features were rejected by fitting a nonlinear SVM model multiple times and at each step removing only one weakest feature. This process is repeated until the model cross-validation score is increased. This subset of features is then combined with statistically significant features to perform stable selection based on subsampling of features in combination with selection algorithm. To conclude with best subsample, we evaluated the proposed model with all possible combinations and then calculating cross-validation scores with average of multiple runs. Thus, we applied three various methods; (Statistical significance, recursive feature elimination and stable selection); of feature selection to select optimal number of best features.

1. **Training and Validating the SVM Model**

Since the number of samples are limited, we used the leave-one-out cross-validation to evaluate the trained model’s performance.

Leave One Out command in Python

*cross_val_score(classifier, X, y, cv= LeaveOneOut() )*

**Equation 3. Developing the nonlinear SVM model**

**Classifier**

SVM intends to maximize the distance between separating hyperplane and support vectors so that classes are best separable. In 2D feature space, separating hyperplane is defined as,

$$y=l.x+d$$

where, $x$ belongs to first class features, while $l$ is the perpendicular distance from support vectors of first class, $d$ is the intercept that belongs to second class features. The cost function of $l$ is given by,

$$S\left( l,\kappa\right)=\frac{1}{2}\left\| l \right\|^{2}+C.\sum_{i=1}^{E} \kappa_{i}$$

Subject to

$\left( l^{T}.x_{i}+d \right)\geq\pm\left( 1-\kappa_{i} \right)$ for $y_{i}=\pm1$, $\kappa_{i}\geq0$

where, $C$ is the regularization parameter between margin and the error, $\kappa_{i}$ is the measure training data error, $E$ is the number of misclassified samples and $y_{i}$ is the class label (Khan et al., 2019). A sigmoid function with $C=0.1$ was applied for classification, while accuracies were obtained using testing data and leave one out cross-validation for training data.

1. **Actual Data Outcomes**

| **Y** | **Condition** | **Number of Subjects** |
| --- | --- | --- |
| 1 | Y ≤ 2 years | 38 |
| 2 | Y > 2 years | 55 |

**Features Selection**

1. **Feature Selection with Statistical Tests for Parametric Data**

**Categorical Features-** Using a test of two proportions

Number of Categorical features using binomial test with P<0.05: 8/30

T2DM; FHx of Cancer; Type of Familial Ca; Bile duct structure; perineural involvement; Margins; Portal tissue resection; Recurrence (Not included in the model)

**Continuous Features-** Using a two sample T-Test

**Number of Continuous features using T-test with P<0.05: 1/20**

Size of Tumor based on MRI (Greatest Length (cm))

1. **Feature Selection with recursive feature elimination and stable selection**

**Categorical Features (9/30)**

T2DM; FHx of Cancer; Type of Familial Ca; Bile duct structure; perineural involvement; Margins; Portal tissue resection; Neoadjuvant; Adjuvant

**Continuous Features (2/20)**

Size of Tumor based on MRI (Greatest Length (cm))

Pathology report: size of tumor (height(cm))

**Comparison of different models**

1. **Using Support Vector Machine**

Training Accuracy for 300 runs: 81.72%

Leave One out cross validation for 300 runs: 75.27%

10% Test data accuracy for 300 runs: 75.26%

Mean Confusion Matrix:

|  |  | Predicted Values | |  |
| --- | --- | --- | --- | --- |
|  |  | Class 1 | Class 2 |  |
| Actual Values | Class 1 | TP=1.6766 | FN=2.3233 | 4 |
|  | Class 2 | FP=0.150 | TN=5.850 | 6 |
|  |  | 1.8266 | 8.1733 |  |

Classification Error: (FP+FN)/Total=24.73%

Sensitivity: TP/(TP+FN) = 41.91%

Specificity: TN/(TN+FP) = 97.5%

1. **Using Random Forest**

Training Accuracy for 300 runs: 79.51%

Leave One out cross validation for 300 runs: 68.44%

10% Test data accuracy for 300 runs: 67.33%

Mean Confusion Matrix:

|  |  | Predicted Values | |  |
| --- | --- | --- | --- | --- |
|  |  | Class 1 | Class 2 |  |
| Actual Values | Class 1 | TP=2.4833 | FN=1.5166 | 4 |
|  | Class 2 | FP=1.75 | TN=4.25 | 6 |
|  |  | 1.766 | 8.233 |  |

Classification Error: (FP+FN)/Total=32.66%

Sensitivity: TP/(TP+FN) = 62.08%

Specificity: TN/(TN+FP) = 70.83%

1. **Using Naïve Bayes**

Training Accuracy for 300 runs: 78.49%

Leave One out cross validation for 300 runs: 75.26%

10% Test data accuracy for 300 runs: 74.69%

Mean Confusion Matrix:

|  |  | Predicted Values | |  |
| --- | --- | --- | --- | --- |
|  |  | Class 1 | Class 2 |  |
| Actual Values | Class 1 | TP=2.97 | FN=1.03 | 4 |
|  | Class 2 | FP=1.5 | TN=4.5 | 6 |
|  |  | 4.47 | 5.53 |  |

Classification Error: (FP+FN)/Total=25.3%

Sensitivity: TP/(TP+FN) = 74.25%

Specificity: TN/(TN+FP) = 75%
