# Supplementary material for: Prognosticating Outcome in Pancreatic Head Cancer With the use of a Machine Learning Algorithm
Source: Technol Cancer Res Treat. 2021 Nov 5;20:15330338211050767. doi: 10.1177/15330338211050767 (PMC8573477; doi:10.1177/15330338211050767)
Supplement: sj-pdf-3-tct-10.1177_15330338211050767 - Supplemental material for Prognosticating Outcome in Pancreatic Head Cancer With the use of a Machine Learning Algorithm [file sj-pdf-3-tct-10.1177_15330338211050767.pdf]

University of Saskatchewan

Certificate of Re-Approval

Application ID:	807
Principal Investigator:	Yigang Luo
Department:	Department of Surgery
Locations Where Research Activities are Conducted:	Royal University Hospital, Canada
Student(s):	Carlos Verdiales<br>Nawaf Abu-Omar<br>Rayyan Khan<br>Ryan Frehlick<br>Zarrukh Baig
Funder(s):	
Sponsor:	
Title:	Predicting Recurrence for Pancreatic Adenocarcinoma Post-Surgical Treatment with use of a Machine Learning Algorithm
Protocol Number:	
Approved On:	23/01/2020
Expiry Date:	22/01/2021
Acknowledgment Of:	
Review Type:	Delegated Review
IRB Registration Number:	Not Applicable

* This study, inclusive of all previously approved documents, has been re-approved until the expiry date noted above

CERTIFICATION

The University of Saskatchewan Biomedical Research Ethics Board (Bio-REB) has reviewed the above-named project.The project is acceptable on scientific and ethical grounds. The principal investigator has the responsibility for any other administrative or regulatory approvals that may pertain to this project, and for ensuring that the authorized project is carried out according to governing law. This approval is valid for the specified period provided there is no change to the approved project.

FIRST TIME REVIEW AND CONTINUING APPROVAL

The University of Saskatchewan Research Ethics Boards review above minimal projects at a full-board (face-to-face) meeting. If a project has been reviewed at a full board meeting, a subsequent project of the same protocol may be reviewed through the delegated review process. Any research classified as minimal risk is reviewed through the delegated (subcommittee) review process. The initial Certificate of Approval includes the approval period the REB has assigned to a study. The Status Report form must be submitted within one month prior to the assigned expiry date. The researcher shall indicate to the REB any specific requirements of the sponsoring organizations (e.g. requirement for full-board review and approval) for the continuing review process deemed necessary for that project.

REB ATTESTATION

In respect to clinical trials, the University of Saskatchewan Research Ethics Board complies with the membership requirements for Research Ethics Boards defined in Part 4 of the Natural Health Products Regulations and Part C Division 5 of the Food and Drug Regulations and carries out its functions in a manner consistent with Good Clinical Practices. Members of the Bio-REB who are named as investigators, do not participate in the discussion related to, nor vote on such studies when presented to the Bio-REB. This approval and the views of this REB have been documented in writing. The University of Saskatchewan Biomedical Research Ethics Board is constituted and operates in accordance with the current version of the Tri-Council Policy Statement: Ethical Conduct for Research Involving Humans (TCPS 2 2018).

Digitally Approved by Gordon McKay, Ph.D.
Chair, Biomedical Research Ethics Board
University of Saskatchewan
